# Supplementary material for: The interactome of intact mitochondria by cross-linking mass spectrometry provides evidence for coexisting respiratory supercomplexes
Source: Mol Cell Proteomics. 2017 Dec 8;17(2):216–32. doi: 10.1074/mcp.RA117.000470 (PMC5795388; doi:10.1074/mcp.RA117.000470)
Supplement: Supplemental Data [file supp_RA117.000470_133922_0_supp_23978_fzffwf.zip › spectra_annotation/mito_DR_spectra_annotation/160-1-8-1-11-1.pdf]

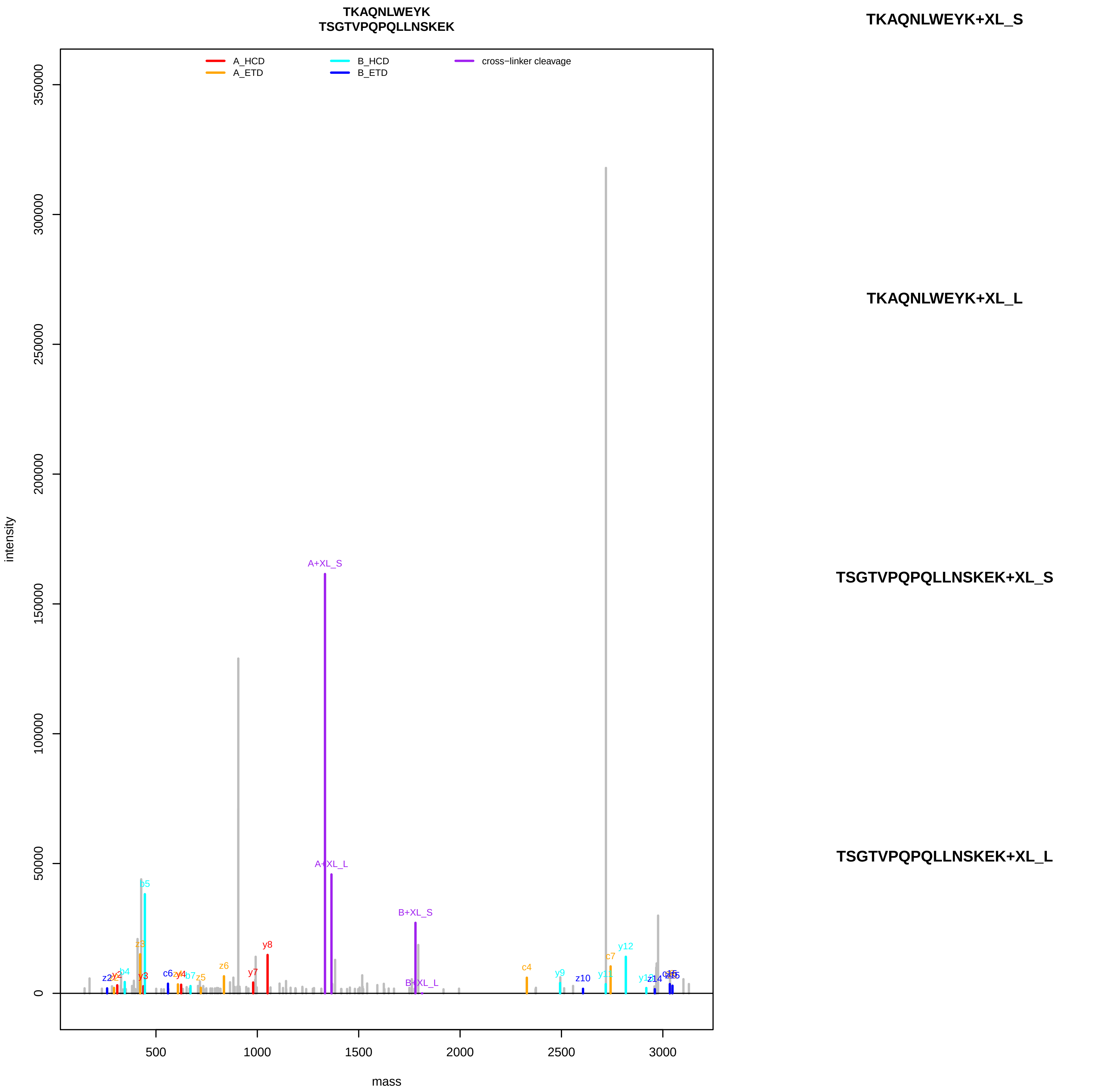

TKAQNLWEYK
TSGTVPQPQLLNSKEK
A_HCD
A_ETD
B_HCD
B_ETD
cross−linker cleavage
intensity
mass
A+XL_S
A+XL_L
B+XL_S
B+XL_L
b5
b4
b7
z3
y3
y8
y7
z6
c6
z5
c4
y9
z10
y12
c7
z14
TKAQNLWEYK+XL_S
TKAQNLWEYK+XL_L
TSGTVPQPQLLNSKEK+XL_S
TSGTVPQPQLLNSKEK+XL_L
